# Martin Salgo and the Dawn of Patient Autonomy

**DOI:** 10.1097/AS9.0000000000000619

**Published:** 2025-10-01

**Authors:** Steven E. Raper

**Affiliations:** From the * Department of Surgery, Perelman School of Medicine, University of Pennsylvania, Philadelphia, PA.

**Keywords:** Autonomy, Nuremberg trials, human experimentation, informed consent, medical malpractice, right to privacy, First amendment

## Abstract

On January 8, 1954, Martin Salgo underwent a translumbar aortogram for a presumptive diagnosis of mid-aortic occlusion. Postprocedure paraplegia occurred, and Mr. Salgo filed a medical malpractice lawsuit claiming, among other causes of action, negligence, breach of a duty to warn, and unauthorized human experimentation. The trial jury initially awarded the plaintiff $250,000. On appeal, Judge Absalom F. Bray reversed the verdict, noting errors of law, specifically, an incorrect theory of negligence. The judge’s opinion highlighted two other important elements of patient autonomy and most famously gave rise to the medicolegal term of art informed consent. But the opinion set out a more nuanced version of a physician and surgeon’s duty to warn than is generally understood. Also highlighted, coming only 5 years after the Nuremberg Doctors’ trials were published in 1949, was clarification of the difference between clinical care and human experimentation. The case of *Salgo v. Leland Stanford Jr. University et al* and its aftermath deserve to be reconsidered as arguably the most important case in medicolegal history and a milestone in advancing the ethical principle of patient autonomy.

## INTRODUCTION

In 1954, a patient at Stanford University Hospital underwent a translumbar aortogram for a presumptive diagnosis of mid-aortic occlusion. Postprocedure paraplegia occurred, leading to a medical malpractice lawsuit claiming, among other causes of action, negligence, breach of a duty to warn, and unauthorized human experimentation. The trial jury initially awarded the plaintiff $250,000. The defendants appealed to the California District Court of Appeals. The appellate judge reversed the verdict, noting an erroneous theory of negligence. The judge’s opinion highlighted 2 other important elements of patient autonomy and most famously gave rise to the medicolegal term of art informed consent. However, the opinion set out a more nuanced version of a physician and surgeon’s duty to warn than is generally understood. Also highlighted, coming only 5 years after the Nuremberg Doctors’ trials publication, was clarification of the difference between clinical care and human experimentation.^1^ The *Salgo* trial and its aftermath deserve to be reconsidered as arguably the most important case in medicolegal history and a milestone in the definition of an ethical right to individual patient autonomy.

## FACTS OF THE CASE

### Medical History

The facts of the case were summarized in the appellate opinion. Martin Salgo was born January 30, 1898, in Hungary to Lewis and Helen Steinberg, for whom records are unavailable. After immigrating to the United States, Mr. Salgo married Olga and had a son, Eugene. Mr. Salgo worked as a shipping clerk at Rand McNally in San Francisco.^2^ At age 55 years, Mr. Salgo was evaluated by Dr. Frank Gerbode for one block claudication, severe back, hip, and right-sided abdominal pain. The exercise intolerance and pain had been progressive over 2 or 3 years. He had some medical treatment without improvement. Past medical history included some eye procedures, suggesting he was older than his stated age. On physical examination, his blood pressure was 180/90. His thighs and calves were atrophic, and his right leg was blue. There was a very weak left femoral pulse, but no right femoral pulse. There were no pulses behind the knee, in the ankles, or in the feet. On raising the legs, they either blanched or turned white. The presumptive diagnosis was occlusion of the abdominal aorta.^3^

**FIGURE 1.**
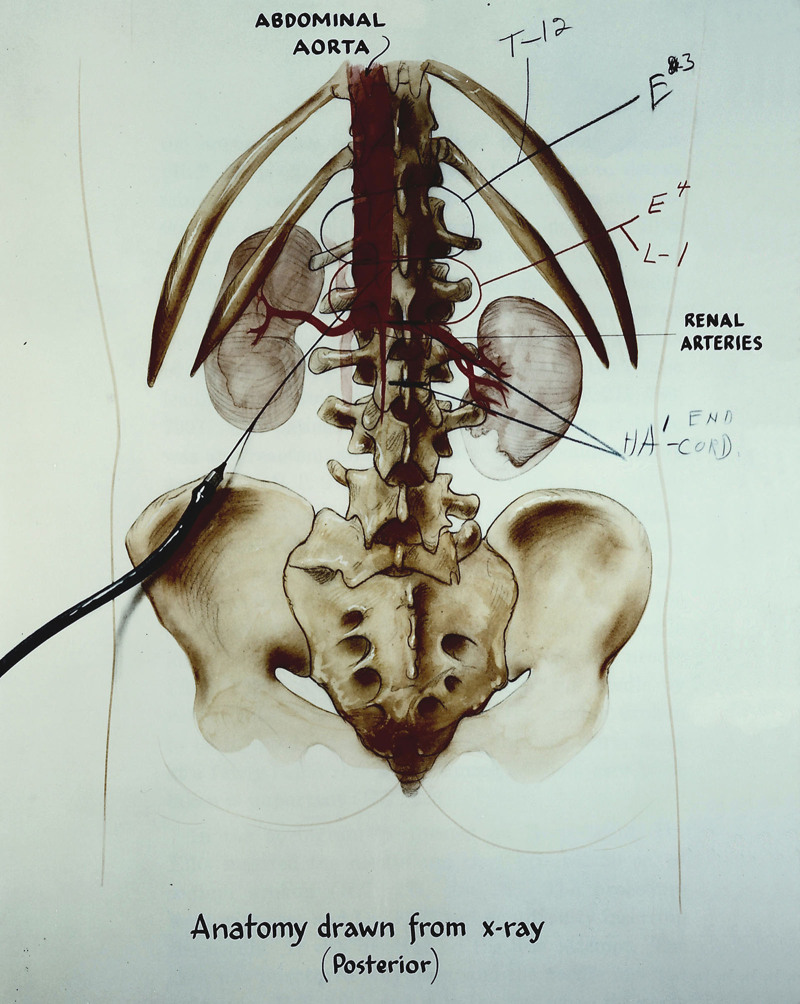
Artist’s representation of Mr. Salgo’s translumbar arteriogram. Note the absence of aortic contrast below the renal arteries. The cause of Mr. Salgo’s paraplegia was never definitively determined.

Mr. Salgo was admitted to Stanford University Hospital, where several hematologic and radiologic studies were performed. On January 8, 1954, a translumbar aortogram was performed by Dr. Eldon E. Ellis. Dr. Ellis, a “surgical fellow”, oversaw all “special diagnostic procedures,” including translumbar aortograms. The study was done under general anesthesia. The translumbar needle entered the aorta in one pass, and 2 injections of iodinated contrast were made. The study confirmed an aortic occlusion just below the renal arteries (Fig. 1). The assessment was that the procedure was routine and everything had gone well. However, on the morning of January 9, the patient was noted to have lower extremity flaccid paralysis and a “cord bladder full of urine”. A neurologist noted areflexia in the legs, a sensory level at the umbilicus to touch, and one level above to pain. There was no proprioception or Babinski reflex.^4^ The record did not state whether systemic anticoagulation was ever provided. Mr. Salgo died in Mt. Zion Hospital in San Francisco on July 19, 1957, without evidence that he ever received an operation. He was cremated and his cremains are interred at the Cypress Lawn Memorial Park in San Francisco, CA.^2^

### Trial Issues and Judgment

The patient and his family sued the Leland Stanford Jr. University Board of Trustees and Stanford University Hospital.^4^ Dr. Frank Gerbode and others were also named in the medical malpractice action. At trial, 2 eminent University of California, San Francisco surgeons were called as expert witnesses; Drs. Edwin Jack Wylie, a vascular surgeon, and Howard C. Nafziger, a neurosurgeon, testified that they could not determine the exact cause of Mr. Salgo’s paraplegia. Three possibilities were discussed: (1) the plaintiff’s condition, a partially blocked aorta, arteriosclerosis, high blood pressure of several years standing, obliteration of blood vessels, and blood supply to the legs meant sudden and total paralysis at any time; (2) direct damage to the spinal cord from Urokon (intravascular contrast) in the spinal cord circulation; (3) constriction of the blood vessels in the spinal cord, due to the Urokon.^5^

The Superior Court of the City and County of San Francisco (California) entered judgment against the defendants, awarding a sum of $250,000 (subsequently adjusted downward to $213,355).^5^ On instruction from the trial judge, a jury found for the plaintiff that the doctrine of *res ipsa loquitur* applied. Loosely translated from Latin, *res ipsa loquitur* means “the thing speaks for itself”. The jury also found that Dr. Gerbode breached a duty to disclose “every risk attendant” upon the surgical procedure.^5^ Further, based on the manufacturer’s brochure, Dr. Gerbode was found to have experimented on the patient.^6^

### Appellate Issues, Holdings, and Subsequent History

On appeal, the judgment for the plaintiff was reversed, not on informed consent, but on a legal theory of *res ipsa loquitur*.^7^ Seven questions were presented: (1) Was *res ipsa loquitur* applicable?; (2) Was Dr. Gerbode liable for negligence of the hospital team?; (3) Were trial court instructions on alleged other negligence of Dr. Gerbode lawful?; (4) Should departing from recommendations in a manufacturer’s brochure be considered experimentation?; (5) Were trial court instructions on the duty to call a specialist, the physician’s duty to disclose, and failure to produce evidence lawful?; (6) Were medical texts properly allowed as evidence?; and (7) Was reference to prior malpractice judgments lawful?^6^

Judge Absalom F. Bray, in writing the opinion, first noted:

“[T]he judgment must be reversed because of error affecting all defendants, namely, improper instructions on res ipsa loquitur. For the guidance of the trial court at the retrial we are considering herein some of the questions raised on this appeal without designation of whether they are raised by all or merely one of the defendants. We do not deem it necessary to discuss all of the questions raised on this appeal, particularly those dealing with other theories of negligence than those discussed herein.”^8^

The doctrine of *res ipsa loquitur* was first recognized in 1945 in the California case of *Ybarra v. Spangard*.^9^ Judge Bray summarized the still relatively new and evolving (in 1954) legal theory of *res ipsa loquitur* as “[W]hen a patient is under anesthesia and a different part of his body is injured than that which should have been involved in the procedure, and there is no evidence that such a result ordinarily might occur without negligence, the doctrine applies.”^10^ In civil trials, the sitting judge or jury is considered the finder of fact, testing evidence for veracity. Such evidence could be based on available documents like medical records, testimony at trial, tangible objects, and expert opinions.^11^ Appellate courts review trial court decisions to ensure that litigants receive justice under the law and consider errors in law (including erroneous jury instructions as in *Salgo*).^12^ Thus, appellate courts are generally limited to correcting errors in law, not errors in fact. Judge Bray held that the trial court had erroneously instructed the jury:

“... that as *a matter of law*, from the ‘happening of all the events involved in this case, however, *as established by the evidence*,’ [emphasis added] there was an inference of negligence. The jury were given no opportunity to determine the facts upon which the doctrine would or would not arise. This was prejudicial error.”^13^

Judge Bray went on to explain that the instructions given to the jury by the trial judge made it impossible to determine whether the jury’s verdict was due to improper *res ipsa loquitur* instructions or other reasons, and the judgment was reversed and remanded for a new trial.^13^ But a petition for a rehearing was denied on November 21, 1957, and Salgo’s petition for a hearing by the California Supreme Court was denied on December 18, 1957, ending the case.

### Error in Instructing on Duty to Warn

There are 2 other facets of the *Salgo* appellate opinion that highlight the importance of the ethical principle of patient autonomy. Although on appeal, Judge Bray reversed due to improper *res ipsa loquitur* jury instructions, he made comments known as *obiter dicta*, or *dicta*. *Dicta* are observations made in judicial opinions that do not form a necessary part of the court’s holding. In *dicta*, the phrase informed consent was introduced into the common law.^14^

Two ethical principles are generally invoked for discussions about informed consent: Beneficence is essentially the “paternalistic doctor who knows what is best for the patient” principle and subsumes 4 elements: do not inflict evil or harm; prevent evil and harm; remove evil and harm; do and promote good.^15^ A second principle is respect for autonomy—persons should be free to choose and act without controlling constraints imposed by others.^16^

It is unclear if the ancients got consent. However, beneficence was the prevailing ethical principle: Hippocrates wrote, “Consider what has gone before, recognize the signs before your eyes and then make your prognosis. Study these principles. Practice two things in your dealings with disease: either help or do not harm the patient.”^17^ Much later, the primacy of the Hippocratic Oath, although silent on consent, was accepted as important in deliberations regarding the Nuremberg Doctors’ Trials.^18^ A leading medieval physician, Henri deMondeville, noted “patients should obey their surgeons implicitly in everything appertaining to their cure.”^19^ During the Enlightenment, Benjamin Rush advocated that “The obedience of a patient to the prescriptions of his physician should be prompt, strict, and universal.” But Rush did want patients sufficiently educated that they could understand the physician’s recommendations as motivation to comply.^20^

In 1847, the American Medical Association published its first code of medical ethics. This code relied heavily on *Medical Ethics* by Thomas Percival. Here again, physician beneficence was the prevailing principle, but did suggest that “to silence a patient with blunt authority may only result in a worsening of the patient’s condition.” Truth telling was not a requirement and lies could be told if the physician thought it was in the best interests of the patient. Percival’s influence persisted in the AMA code up until the 1980s.^21^

The foundation of the current consent process is active patient participation in decision-making. The consent process integrates an ongoing conversation between physician and patient as a routine part of diagnosis and treatment. Consent as a process is not an arbitrary legal imposition on clinical care or a ritual that the patient cannot understand. The event model inserts the informed consent document into decision-making by the surgeon, recalling the beneficence principle.^22^

There are 2 theories of liability under the law: negligence and battery. Medical malpractice is a special case of the tort of negligence and is the cause of action in most jurisdictions. One needs to show a duty, breach of that duty, proximate cause, and damages.^23^ Complete absence of consent is generally considered a medical battery, or unauthorized touching. In a claim of battery, one need only show duty and breach.^24^ Why are surgeons compelled to provide informed consent? Although the First Amendment of the Bill of Rights has protections against compelled speech, the U.S. Supreme Court has affirmed the requirement that a doctor obtain informed consent to perform an operation as “firmly entrenched in American tort law.”^25^ Further, the law regulates speech only “as part of the practice of medicine, subject to reasonable licensing and regulation by the State.”^26^

A long line of common law legal decisions has refined the consent doctrine. The leading cases are briefly mentioned here; most, if not, all have been codified in state courts or legislation. Consent did not always have to be informed. In Mohr, the surgeon got consent for right ear surgery, but the pathology was on the left, and surgery was performed. The court found the surgeon liable for assault and battery, and “has no free license respecting surgical operations.”^27^ In *Pratt v. Davis*, Davis, one of the founders of the American College of Surgeons, was found liable for performing a hysterectomy without consent.^28^ In *Rolater v. Strain*, the patient agreed to drainage of a foot infection, but specifically stated no bone was to be removed. The surgeon did remove bone, and the Court found the surgeon liable for assault and battery.^29^

In *Schloendorff v. Society of New York Hospitals*, the patient agreed to an exam under anesthesia, but no operation. The surgeon removed a giant fibroid tumor, and the patient sued. Benjamin Nathan Cardozo uttered one of the most famous pronouncements in medicolegal jurisprudence: “Every human being of adult years and sound mind has a right to determine what shall be done with his own body; and a surgeon who performs an operation without his patient’s consent commits an assault, for which he is liable in damages.”^30^

Professional societies also began to weigh in on the contours of consent. The American Board of Surgery in 1956 (after the original *Salgo* decision in 1954) noted that informed consent was “a moral and legal prerequisite” for surgery.^31^ In 1957, the American Medical Association published a consent to operations and other procedures stating, “The consent given must be an informed consent with an understanding of what is to be done and of the risks involved.”^31^ The American College of Surgeons, in its initial statement, cautioned the surgeon to obtain written consent.^31^

One other important facet of individual autonomy is the constitutional right to privacy.^32^ Perhaps the most eloquent statement of individual autonomy as regards the right to privacy is that of Justice Douglas, “[T]he freedom to care for one’s health and person, freedom from restraint or compulsion, freedom to walk, stroll, or loaf.”^33^ There are 2 important advantages to constitutional claims, despite the fact that the Constitution only protects individuals against state action.^34^ First, informed consent statutes can be challenged in court.^34^ An even greater strength for constitutional claims is the power to order parties to act—or not act—prospectively unlike the common law of informed consent, where patients can only sue for money damages after an injury has occurred.^34^

Informed consent, uttered for the first time in the context of clinical medicine makes *Salgo v. Leland Stanford Jr. University Board of Trustees* another of the most important cases in medical consent law.^7^ In fact, one review between 1930 and 1956 showed only 9 published articles on consent.^35^ At trial, the judge gave a jury instruction (#32 of 78!) that stated:

“[I]t is the duty of a physician and surgeon who has undertaken the care and treatment of a patient to exercise the highest degree of good faith in dealing with his patient and to this end owes a duty to make a full disclosure of *all* [emphasis added] the facts which mutually affect his rights and interests and of the surgical risk, hazard and danger, if any...”^36^

On appeal, Judge Bray noted “The [trial] court gave a rather broad instruction upon the duty of a physician to disclose to the patient ‘all the facts...’”^7^ Further, in *dicta*, “The instruction given should be modified to inform the jury that the physician has such discretion consistent, of course, with the full disclosure of facts necessary to an *informed consent*.”^7^ Although Judge Bray is properly credited with using informed consent in his appellate opinion, this phrase seems to have been lightly edited from an amicus curiae brief filed by the American College of Surgeons and written by 2 attorneys, Lawrence Howe, Jr. and Paul G. Gebhard.^37^ Although widely acknowledged as the first to coin the term in medical jurisprudence, the phrase informed consent had been used in other settings before *Salgo*.^31^

Salgo balanced what subsequently became 2 leading contemporary consent standards: the professional practice standard and the reasonable person standard.^38^ In mandating a change in the jury instruction, Judge Bray said a physician, while placing the patient’s welfare above all else, must sometimes either choose between explaining all risks about an invasive procedure or operation, no matter how remote, or alternatively, to recognize that each patient’s understanding and emotional make-up is unique.^7^ One example would be an individual patient, already anxious about meeting a surgeon, becoming alarmed and refusing to consent to an operation with otherwise acceptable risks.^7^

The earliest standard, professional practice, states that the duty to disclose is determined by customary practices of the professional community.^38^ *Natanson v. Kline*, citing *Salgo*, was among the first to articulate the professional practice standard, holding, “The duty of the physician... is limited to those disclosures which a reasonable medical practitioner would make under the same or similar circumstances.”^39^ Subsequent principal objections to the professional practice standard include leaving the extent of disclosure to physicians and surgeons undermines patient autonomy, whether local or national standards should apply, and the extent to which consensus is required to set a professional standard.^40^

Seemingly at odds with the need for physicians and surgeons to balance risks against the patient’s mental and emotional needs, Judge Bray in *Salgo* goes on to say, “A physician violates his duty to his patient and subjects himself to liability if he withholds *any facts* [emphasis added] which are necessary to form the basis of an intelligent consent.”^7^ Thus, a second standard arose to counter objections to professional practice, that of the reasonable person.^41^ *Canterbury v. Spence*, a famous case setting forth the reasonable person standard, states “[A] reasonable person, in what the physician knows or should know to be the patient’s position, would be likely to attach significance to the risk or cluster of risks in deciding whether or not to undergo the proposed therapy.”^42^ *Canterbury* also cites *Salgo* for the concerns about patients as unique individuals as a possible exception, if only to delineate boundaries: “The critical inquiry... is whether the physician responded to a sound medical judgment that communication of the risk information would present a threat to the patient’s well-being.”^43^

Judge Bray contemplated the need for physicians and surgeons to balance the risks of a procedure, stating that each patient “presents a separate problem, that the patient’s mental and emotional condition is important...”^7^ That an abstract “reasonable person” does not account for the facts specific to a real patient led some commentators to propose a third standard, that of subjective disclosure, requiring a surgeon to disclose information specific to an individual patient.^21^ Advocates of the subjective standard claim that the right to make decisions for personal reasons is not adequately protected by the other commonly accepted standards.^44^ Critics of the subjective standard argue “... it places an unfair legal burden on physicians to intuit the idiosyncratic values and interests of their patients, [leaving physicians] at the mercy of their patients’ self-serving hindsight in court.”^45^ If patients have the right to make decisions for personal reasons, including certain medical comorbidities, such reasons might not be considered material by a reasonable person, however defined. Most courts have articulated reasons for not using the subjective standard.^46^ A subjective standard puts the physician in the position of entering the patient’s mind, including, for example, disclosure of any comorbidities, that if not completely illuminated, could be adjudged a failure to disclose material facts.^46^

## DISTINGUISHING CLINICAL CARE FROM HUMAN EXPERIMENTATION

A second important feature of *Salgo* arose in the context of *The Medical Case* before the Nuremberg Military Tribunals and was widely reported in the United States.^47–49^ The atrocities inflicted on Nazi victims and other emerging reports of research subject abuse “raised suspicions about the general trustworthiness of the medical profession.”^50^ .

The Nuremberg “Medical Case” trial charged horrifying examples of unethical human “experiments” as crimes against humanity.^51^ The Nuremberg *Medical Case* trial concluded that the interests of physicians and patients are not always aligned, especially in medical experimentation.^52^ Nazi physicians were tried, convicted, and sentenced for the barbarism and murder of concentration camp inmates who “volunteered” during “medical experiments.”^53^ The German Reich had issued “guidelines for new therapy and human experimentation” that were ignored by the Nazi “physicians.”^54^ Recognizing that the United States did not have written guidelines, the Council of the American Medical Association—in the Principles of Medical Ethics—approved for human experimentation 3 requirements as promulgated by Dr. Andrew C. Ivy: (1) the individual on whom the experiment is to be performed must give voluntary consent, (2) animal experimentation should first be done to assess dangers of the experiment, and (3) the experiment must be performed under proper medical protection and management.^55^ These rules were adopted by the American Medical Association House of Delegates and introduced into the record of the Nuremberg Trials as generally accepted practice.^56^ The Nuremberg tribunal went further by promulgating 10 basic principles for the conduct of human subject research, including the role of voluntary consent, subsequently known as the Nuremberg Code (Table 1).^57^

**TABLE 1.** Directives for Human Experimentation of the Nuernberg Code^34^

1. Voluntary consent of the human subject.
2. Experiments should yield fruitful results for the good of society.
3. Anticipated results should justify the experiment.
4. Researchers should avoid unnecessary physical and mental suffering.
5. No experiment should be done if there is reason to think that death or disabling injury will occur.
6. Risks should not exceed the experiment’s humanitarian importance.
7. Experimental subjects should be protected against the remotest possibilities of injury, disability, or death.
8. Only scientifically qualified persons should conduct experiments.
9. Subjects should be free to bring the experiment to an end.
10. The researcher should terminate the experiment at any stage if continuation is likely to result in injury, disability, or death.

The Nuremberg Trials were published a mere 5 years before *Salgo* was litigated.^58^ The Nuremberg Code, with major contributions by Drs. Andrew C. Ivy and Leo Alexander were intended not only to prevent human rights atrocities such as those perpetrated by the Third Reich, but also to ensure future performance of ethical human subjects research.^59,60^ Jay Katz, a noted bioethicist, observed that the Nuremberg tribunal went to great lengths to define voluntary consent, including the subject’s capacity to give consent.^61^ The Nuremberg Code was accepted as “the one document that seeks in uncompromising language to protect the inviolability of subjects of research.”^62^ And, in time, the Nuremberg Code was adopted in some form by most countries of the world, and eventually additional restraints were added.^63^

In *Salgo*, an additional claim of unconsented human experimentation was leveled at the defendants.^64^ The charge stemmed from the use of more Urokon than recommended in a manufacturer’s brochure, and 2 injections were necessary to obtain optimum diagnostic data. Instruction 42 was given to the jury “[I]f and when a physician and surgeon seeks fields of experimentation... he will be accountable for any damages proximately caused by any unskillful treatment...”^65^ And the trial jury also received Instruction 46 “... if you further find that such injection [Urokon] constituted experimenting in the performance of the operation..”^66^ The appellate opinion held that a departure from a manufacturer’s recommendation does not constitute an experiment and instructions on experimentation should not have been given. Again, in *dicta*, Judge Bray opined:

“[Appellants] contend that the miraculous developments which have taken place in the effective use of antibiotics and other drugs might never have been accomplished if physicians were required to follow blindly the suggestions of the manufacturers who prepare but do not use them... There was in this case no evidence of experiment and the instructions concerning ‘experiment’ should not have been given.”^67^

Years after *Salgo* was decided, the United States finally expanded and refined the Nuremberg Code. The National Research Act of 1974 formed the National Commission for the Protection of Human Subjects of Biomedical and Behavioral Research (The Belmont Commission).^68^ The role of the Belmont Commission was to articulate the role of research subject autonomy in The Belmont Report, a seminal document consisting of 3 guiding principles by which human subjects research should be performed: respect for persons, beneficence, and justice.^69^ As did *Salgo*, the Belmont Report was careful to draw distinctions between medical practice and clinical research; “Medical...practice is to provide diagnosis, preventive treatment or therapy.... [T]he term ‘research’ designates an activity designed to test an hypothesis, permit conclusions to be drawn, and thereby to develop or contribute to generalizable knowledge...”^70^ Additional important statements on ethical clinical research and the role of informed consent include the World Medical Association’s Declaration of Helsinki, the Geneva Declaration, and the International Code of Medical Ethics.^71^

## SUMMARY AND CONCLUSIONS

In considering the work of Freud, the psychoanalyst Harry Guntrip opined, “[There is a] permanent place in the history of thought that belongs to the genuine pioneer. It is not the function of the pioneer to say the last word but to say the first word.”^72^ In the annals of medicolegal jurisprudence, Judge Bray should be considered such a pioneer. He began with existing problems and looked at them anew in a particularly illuminating way. Although *res ipsa loquitur*, patient consent, and the special concerns about human experimentation were all present before *Salgo*, Judge Bray’s thoughtful analysis represented the beginning of the definition of an ethical right to individual patient autonomy—a tipping point, if you will—that continues to evolve to this day. We ignore the lessons of *Salgo* for contemporary analyses of the primacy of patient autonomy at our peril.
